# Detection and characterization of bacterial endosymbionts in Southeast Asian tephritid fruit fly populations

**DOI:** 10.1186/s12866-019-1653-x

**Published:** 2019-12-24

**Authors:** Elias D. Asimakis, Vangelis Doudoumis, Ashok B. Hadapad, Ramesh S. Hire, Costas Batargias, Changying Niu, Mahfuza Khan, Kostas Bourtzis, George Tsiamis

**Affiliations:** 1 Department of Environmental Engineering, University of Patras, 2 Seferi St., 30100 Agrinio, Greece; 2 Department of Fisheries & Aquaculture Management, Technological Educational Institute of Western Greece, 30200 Messolonghi, Greece; 3 Nuclear Agriculture & Biotechnology Division, Bhabha Atomic Research Centre (BARC), Trombay, Mumbai, Maharashtra 400 085 India; 4 Huazhong Agricultural University, Wuhan, 430070 Hubei China; 5 Insect Biotechnology Division, Institute of Food and Radiation Biology (IFRB), Atomic Energy Research Establishment (AERE), Ganakbari, Savar, Dhaka 1349 Bangladesh; 6 Insect Pest Control Laboratory, Joint FAO/IAEA Division of Nuclear Techniques in Food and Agriculture, Vienna International Centre, P.O. Box 100, 1400 Vienna, Austria

**Keywords:** 16S *rRNA*, Multi locus sequence typing, *Wolbachia*, *Arsenophonus*, *Cardinium*, *Spiroplasma*, Horizontal gene transfer, *Bactrocera*, *Zeugodacus*

## Abstract

**Background:**

Various endosymbiotic bacteria, including *Wolbachia* of the Alphaproteobacteria, infect a wide range of insects and are capable of inducing reproductive abnormalities to their hosts such as cytoplasmic incompatibility (CI), parthenogenesis, feminization and male-killing. These extended phenotypes can be potentially exploited in enhancing environmentally friendly methods, such as the sterile insect technique (SIT), for controlling natural populations of agricultural pests. The goal of the present study is to investigate the presence of *Wolbachia*, *Spiroplasma*,*Arsenophonus* and *Cardinium* among *Bactrocera*,*Dacus* and *Zeugodacus* flies of Southeast Asian populations, and to genotype any detected *Wolbachia* strains.

**Results:**

A specific 16S *rRNA* PCR assay was used to investigate the presence of reproductive parasites in natural populations of nine different tephritid species originating from three Asian countries, Bangladesh, China and India. *Wolbachia* infections were identified in *Bactrocera dorsalis*, *B. correcta*, *B. scutellaris* and*B. zonata*, with 12.2–42.9% occurrence, Entomoplasmatales in *B. dorsalis*, *B. correcta*, *B. scutellaris*, *B. zonata*,*Zeugodacus cucurbitae* and *Z. tau* (0.8–14.3%) and *Cardinium* in *B. dorsalis* and*Z. tau* (0.9–5.8%), while none of the species tested, harbored infections with *Arsenophonus*. Infected populations showed a medium (between 10 and 90%) or low (< 10%) prevalence, ranging from 3 to 80% for *Wolbachia*, 2 to 33% for Entomoplasmatales and 5 to 45% for *Cardinium*. *Wolbachia* and Entomoplasmatales infections were found both in tropical and subtropical populations, the former mostly in India and the latter in various regions of India and Bangladesh. *Cardinium* infections were identified in both countries but only in subtropical populations. Phylogenetic analysis revealed the presence of*Wolbachia* with some strains belonging either to supergroup B or supergroup A. Sequence analysis revealed deletions of variable length and nucleotide variation in three *Wolbachia* genes. *Spiroplasma* strains were characterized as citri–chrysopicola–mirum and ixodetis strains while the remaining Entomoplasmatales to the Mycoides–Entomoplasmataceae clade.*Cardinium* strains were characterized as group A, similar to strains infecting *Encarsia pergandiella*.

**Conclusions:**

Our results indicated that in the Southeast natural populations examined, supergroup A *Wolbachia* strain infections were the most common, followed by Entomoplasmatales and *Cardinium*. In terms of diversity, most strains of each bacterial genus detected clustered in a common group. Interestingly, the deletions detected in three *Wolbachia* genes were either new or similar to those of previously identified pseudogenes that were integrated in the host genome indicating putative horizontal gene transfer events in *B. dorsalis*, *B. correcta* and *B. zonata*.

## Background

In recent years, many maternally inherited endosymbiotic bacteria, capable of manipulating the reproductive functions of their hosts, have been identified in a wide range of arthropod species [1]. Among them, the most thoroughly studied are those that belong to the genus *Wolbachia*, a highly diverse group of intracellular endosymbionts belonging to the Alphaproteobacteria [2–4]. *Wolbachia* infections are widespread in insect species with estimates suggesting an incidence rate ranging from 20 to 66% [5–10]. *Wolbachia* infections vary significantly between species and also between different geographical populations of a species, exhibiting either high (> 90%) or low prevalence (< 10%) [5, 11, 12]. Overall, the diverse interactions of *Wolbachia* with their hosts cover a broad spectrum of biological, ecological and evolutionary processes [13–17]. One of the most interesting aspects of *Wolbachia* interactions is the induction of a range of reproductive abnormalities to their hosts, such as cytoplasmic incompatibility (CI), parthenogenesis, male-killing and feminization of genetic males so they develop as females [3, 14, 18–20]. For instance, in woodlice, genetic males develop as females when *Wolbachia* disrupts a gland that produces a hormone required for male development [21]. In this way, the bacteria change the birth ratio in favor of females, ensuring their steady proliferation within host populations, since they are vertically transmitted by infected females [2, 3, 17, 20, 22].

Apart from *Wolbachia*, additional reproductive symbionts from distantly related bacterial genera have been recently brought to light, such as *Arsenophonus*, *Cardinium* and *Spiroplasma*. Strains belonging to the genus *Cardinium*, a member of the phylum Cytophaga-Flavobacterium-Bacteroides (CFB), exhibit the same broad range of reproductive alterations with *Wolbachia* [23–29], with the exception of male-killing which has not been identified yet [1, 17, 28]. On the other hand, members of *Arsenophonus*, of the Gammaproteobacteria, and *Spiroplasma*, wall-less bacteria belonging to the class Mollicutes, are known to induce male-killing phenotypes [1, 17, 30–32]. The incidence rate of all three genera in insects was shown to vary between 4 and 14%, fairly lower than that of *Wolbachia* [1, 33–39], although higher occurrence was observed for *Arsenophonus* in aphids and ants, reaching up to 30 and 37.5% of species respectively [40, 41] as well as for *Cardinium* in planthoppers (47.4% of species) [36]. In *Cardinium* and *Spiroplasma*-infected species a wide range of prevalence (15–85%) was observed while in the case of *Arsenophonus*, prevalence reached values above 75% with relatively few exceptions, such as the wasp *Nasonia vitripennis* with a 4% infection rate or various ant species that showed a broader range (14–66%) [1, 38, 40, 42].

Insect species belonging to the genus *Bactrocera* and the closely related species *Dacus longicornis* (Wiedemann), *Z*.*cucurbitae* (Coquillett) and *Z. tau* (Walker) are members of the Tephritidae, a family of fruit flies with worldwide distribution that contains important agricultural pests, capable of affecting a variety of fruit and horticultural hosts [43–46]. The direct damage to hosts caused by female oviposition and the development of the larvae, results in severe losses in fruit and vegetable production. Their economic impact also expands to trade, with strict quarantine measures imposed on shipments originating from infested countries [47–50]. The reproductive alterations induced by the bacterial symbionts, as well as their role in insect host biology and ecology, could be used in environment-friendly approaches, such as the sterile insect technique (SIT) and other related techniques, for the area-wide integrated pest management (AW-IPM) of insect pest populations [13, 51–65].

The current classification of *Wolbachia* strains based on molecular markers includes 16 supergroups, from A to Q, with the exception of G which has been merged with A and B [66–71]. Classification is primarily based on the 16S *rRNA* gene but other commonly used genetic markers include the *gltA* (citrate synthase), *groEL* (heat-shock protein 60), *coxA* (cytochrome c oxidase), *fbpA* (fructose-bisphosphatealdolase),*ftsZ* (cell division protein), *gatB* (glutamyl-tRNA(Gln) amidotransferase, subunit B),*hcpA* (hypothetical conserved protein) and*wsp* genes (*Wolbachia* surface protein) [7, 72, 73]. Strain genotyping is performed by multi locus sequence typing (MLST) using five conserved genes (*coxA*, *fbpA*, *ftsZ*, *gatB* and*hcpA*), the *wsp* gene and four hypervariable regions (HVRs) of the WSP protein [74]. Similarly, *Spiroplasma* strains are divided into three groups, the apis clade, the citri–chrysopicola–mirum clade and the ixodetis clade [75, 76]. Phylogenetic analyses are primarily based on the 16S*rRNA* gene, while more detailed MLST approaches include partial sequencing of the 23S *rRNA*, 5S *rRNA*, *gyrB*, *rpoB*,*pgk* (phosphoglycerate kinase) *parE*, *ftsZ, fruR* genes, as well as the complete 16S–23S internal transcribed spacer region (ITS) [75, 77]. The remaining closely related Entomoplasmatales genera,*Mycoplasma*, *Entomoplasma* and *Mesoplasma*, form the separate Mycoides–Entomoplasmataceae clade [76]. Phylogenetic analyses for *Cardinium* are performed with the use of the 16S *rRNA* and *gyrB* genes but also with the amino acid sequence of Gyrase B (*gyrB* gene) [35, 36, 78–80]. *Cardinium* strains can be separated into group A, which infect wasps, planthoppers, mites and other arthropods, group B, found in parasitic nematodes and group C in biting midges [36].

Several studies reported that genes, chromosomal segments of various sizes or even the entire *Wolbachia* genome have been horizontally transferred to host chromosomes [81, 82]. The first incidence of a horizontal gene transfer (HGT) event was described in the adzuki bean beetle *Callosobruchus chinensis* (L.), where ~ 30% of the *Wolbachia* genome was found to be integrated in the X chromosome [83, 84]. Such events have also been described in a variety of insect and nematode hosts, including the fruit fly*Drosophila ananassae* and the tsetse fly*Glossina morsitans morsitans* [81, 85–89]. In *G. m. morsitans* two large *Wolbachia* genome segments of 527 and 484 Kbp have been integrated into the *Gmm* chromosomes, corresponding to 51.7% and 47.5.% of the draft *Wolbachia* genome [90]. In the case of *Drosophila ananassae*, nearly the entire ~ 1.4 Mbp*Wolbachia* genome has been integrated in a host chromosome [81] while in *Armadillidium vulgare* the ~ 1.5 Mbp *Wolbachia* genome was not only integrated but also duplicated, resulting in the formation of a new female sex chromosome [91]. In the case of the mosquito *Aedes aegypti*, the direction of the HGT is not clear and could have happened either from the insect or from *Wolbachia* [92, 93]. Usually, the incorporated fragments lose their functionality and become pseudogenes with low levels of transcription [88]. However, some of these genes are highly expressed and can either provide a new function to the host, or replace a lost one [89, 92, 93]. These new functions may provide hosts with nutritional benefits, enable them to parasitize other eukaryotes, survive in unfavorable environments or protect themselves from other organisms [88].

In the present study, we investigate the presence of *Wolbachia*, *Cardinium* and Entomoplasmatales (the genera *Spiroplasma*,*Entomoplasma* and *Mesoplasma*) infections in natural populations of *Bactrocera*, *Dacus* and *Zeugodacus* fruit fly species. The detection and the phylogenetic analysis of the bacterial genera were based primarily on the use of the 16S *rRNA* gene. Additionally, the molecular characterization of the *Wolbachia* strains was performed with the use of the *wsp* and MLST gene markers. Finally, we report on the presence of *Wolbachia* pseudogenes suggesting putative horizontal transfer events to the genome of various *Bactrocera* species and*Z*. *cucurbitae*.

## Results

### Infection prevalence of reproductive symbiotic bacteria

*Wolbachia*, Entomoplasmatales and*Cardinium* infections were detected in 15 populations, divided into six species of *Bactrocera* and *Zeugodacus* (Tables 1, 2). *Wolbachia* was the most prevalent with 64 out of 801 (8%) infected individuals, followed by 40 (5%) Entomoplasmatales and 12 (1.5%) *Cardinium* (Tables 1 and 2). On the contrary, no *Arsenophonus* infections were found in any of the populations tested. *Bactrocera minax* (Enderlein),*B. nigrofemoralis* (White & Tsuruta) and *D. longicornis* were the only species that did not harbor any infections of the bacterial symbionts tested in this study (Table 2).

**Table 1:** Prevalence of reproductive bacteria in tephritid fruit fly populations from Bangladesh, China and India using a 16S *rRNA* gene-based PCR screening approach. For each genus the absolute number and the percentage (in parentheses) of infected individuals are given. The last column on the right (“Total*”) indicates the total occurrence of all three Entomoplasmatales genera

								Entomoplasmatales			
	Species	Country	State	Area	Samples	*Wolbachia*	*Cardinium*	*Spiroplasma*	*Entomoplasma*	*Mesoplasma*	Total*
1	*B. correcta*	India	Maharashtra	Trombay	25	10 (40)	0	0	1 (4)	0	1 (4)
2	*B. correcta*	India	Karnataka	Raichur	5	0	0	0	0	0	0
3	*B. dorsalis*	Bangladesh	–	Rajshahi	36	1 (2.8)	0	0	6 (16.7)	0	6 (16.7)
4	*B. dorsalis*	Bangladesh	–	–	29	0	0	0	0	0	0
5	*B. dorsalis*	Bangladesh	–	Dinajpur	22	0	10 (45.5)	0	0	0	0
6	*B. dorsalis*	Bangladesh	–	Dhaka	34	0	0	0	0	0	0
7	*B. dorsalis*	Bangladesh	–	Jessore	23	0	0	0	0	0	0
8	*B. dorsalis*	India	Maharashtra	Trombay	30	14 (46.7)	0	2 (6.7)	5 (16.7)	0	7 (23.3)
9	*B. dorsalis*	India	Himachal Pradesh	Palampur	15	10 (66.7)	1 (6.7)	0	5 (33.3)	0	5 (33.3)
10	*B. minax*	China	–	–	40	0	0	0	0	0	0
11	*B. nigrofemoralis*	India	Himachal Pradesh	Palampur	5	2^a^ (0)	0	0	0	0	0
12	*B. scutellaris*	India	Himachal Pradesh	Palampur	35	15 (42.9)	0	0	5 (14.3)	0	5 (14.3)
13	*B. zonata*	Bangladesh	–	Rajshahi	21	2^a^ (0)	0	0	2 (9.5)	2 (9.5)	4 (19)
14	*B. zonata*	Bangladesh	–	Jessore	33	0	0	0	0	0	0
15	*B. zonata*	Bangladesh	–	Dinajpur	26	0	0	0	0	0	0
16	*B. zonata*	India	Maharashtra	Trombay	25	10 (40)	0	0	3 (12)	0	3 (12)
17	*B. zonata*	India	Karnataka	Raichur	5	4 (80)	0	0	0	1 (20)	1 (20)
18	*B. zonata*	India	Himachal Pradesh	Palampur	5	0	0	0	1 (20)	0	1 (20)
19	*D. longicornis*	Bangladesh	–	Dhaka	21	0	0	0	0	0	0
20	*Z. cucurbitae*	Bangladesh	–	Rajshahi	47	0	0	0	0	0	0
21	*Z. cucurbitae*	Bangladesh	–	Jessore	55	0	0	0	0	1 (1.8)	1 (1.8)
22	*Z. cucurbitae*	Bangladesh	–	–	30	0	0	0	0	0	0
23	*Z. cucurbitae*	Bangladesh	–	Dinajpur	96	2^a^ (0)	0	1 (1)	2 (2.1)	0	3 (3.1)
24	*Z. cucurbitae*	Bangladesh	–	Dhaka	29	0	0	0	2 (6.9)	0	2 (6.9)
25	*Z. tau*	Bangladesh	–	Jessore	22	0	0	0	0	0	0
26	*Z. tau*	Bangladesh	–	Dhaka	6	0	0	0	0	0	0
27	*Z. tau*	Bangladesh	–	Rajshahi	31	0	0	0	0	0	0
28	*Z. tau*	Bangladesh	–	Dinajpur	20	0	1 (5)	0	0	0	0
29	*Z. tau*	India	Maharashtra	Trombay	10	0	0	0	0	1 (10)	1 (10)
30	*Z. tau*	India	Himachal Pradesh	Palampur	20	0	0	0	0	0	0
Total	9	3	3	7	801						

^a^. Only pseudogenised sequences

**Table 2:** Prevalence of reproductive symbionts in different tephritid fruit fly species

						Entomoplasmatales		
Species	Country	Areas with infected populations	Samples	*Wolbachia*	*Cardinium*	*Spiroplasma*	*Entomoplasma*	*Mesoplasma*
*B. correcta*	India	Trombay	30	10^b^ (30%)	–	–	1 (3.3%)	–
*B. dorsalis*	India	Trombay, Palampur	189	25^b^ (13.2%)	11 (5.8%)	2 (1.1%)	16 (8.5%)	–
	Bangladesh	Rajshahi, Dinajpur						
*B. minax*	China	–	40	–	–	–	–	–
*B. nigrofemoralis*	India	Palampur	5	2^a^ (0%)	–	–	–	–
*B. scutellaris*	India	Palampur	35	15 (42.9%)	–	–	5 (14.3%)	–
*B. zonata*	India	Trombay, Raichur, Palampur	115	14^b^ (12.2%)	–	–	6 (5.2%)	3 (2.6%)
	Bangladesh	Rajshahi		2^a^ (0%)				
*D. longicornis*	Bangladesh	Dhaka	21	–	–	–	–	–
*Z. cucurbitae*	Bangladesh	Dinajpur, Jessore, Dhaka	257	2^a^ (0%)	–	1 (0.4%)	4 (1.6%)	1 (0.4%)
*Z. tau*	India	Trombay	109	–	1 (0.9%)	–	–	1 (0.9%)
	Bangladesh	Dinajpur						

^a^. Only pseudogenized sequences

^b^. Both integral (genuine or full) and pseudogenized *Wolbachia* genes

The presence of *Wolbachia*, at variable infection rates, was identified in seven populations from four different species of tephritid fruit flies (Table 2). The most prevalent infections were observed in *B. scutellaris* (Bezzi) (42.9%) and *B. correcta* (Bezzi) (30%) compared to *B. dorsalis* (Hendel) (13.2%) and *B. zonata* (Saunders) (12.2%) (chi-squared test:*p*-values< 0.01). On the other hand, no*Wolbachia* infections were identified in the remaining species tested, namely, *D. longicornis*, *B. minax*,*B. nigrofemoralis*, *Z. cucurbitae* and *Z. tau*. Variation in prevalence was observed between field populations of the same species from different geographic regions. For example,*Wolbachia* infections in *B. zonata* were characterized by 80% prevalence in a population from Raichur, India, by 40% in Trombay, India and were absent from the remaining four areas tested (Table 1, Additional file 1). Heterogeneity in infection rates was also observed in *B. dorsalis*, which showed medium prevalence (46.7 and 66.7%), except for a population from Rajshahi – the only infected population from Bangladesh – which showed a considerably lower infection rate (2.8%) (chi-squared test:*p*-values< 0.01). The remaining four*B. dorsalis* populations appeared to be free of *Wolbachia* infections. Only one of two*B. correcta* populations studied was infected with *Wolbachia*, the population originating from the area of Trombay, India with 40% prevalence. Finally, in the case of *B. scutellaris*, the only population tested was found to be infected at 42.9% rate. *Wolbachia* prevalence also ranged significantly between populations of the same species that originated from different countries, with fruit flies from India exhibiting higher infection rate than those from Bangladesh. More specifically, Indian populations of *B. dorsalis* and *B. zonata* exhibited 53.3 and 40% prevalence respectively, significantly higher than populations from Bangladesh that were found to contain only 0.7% and pseudogenized *Wolbachia* sequences respectively (chi-squared test: *p*-values< 0.01) (Table 1).

The occurrence of *Spiroplasma* and its relative genera, *Entomoplasma* and*Mesoplasma*, displayed variation between different species, populations and countries (Tables 1, 2). Again, the most prevalent infections per species were observed in *B. scutellaris* (14.3%) followed by *B. dorsalis* (9.6%) and *B. zonata* (7.8%). Three more species were infected with members of the Entomoplasmatales, including *B. correcta* (3.3%), and at much lower rate compared to the three species with prevalent infections, *Z. cucurbitae* (2.4%) and *Z. tau* (0.9%) (chi-squared test: *p*-values< 0.01). The remaining species that were tested, including *B. minax*, *B. nigrofemoralis* and *D. longicornis*, appeared to be free of Entomoplasmatales infections (Table 2). In some cases, the infection rate varied between different populations. For example, in *B. dorsalis*, prevalence ranged from 33.3% in Palampur, to 23.4% in the Trombay area, in India and 16.7% in the Rajshahi District, in north-western Bangladesh. There were also four populations from Bangladesh that did not contain any infections (Table 1). At the same time, *B. zonata* infection rates were almost uniform in three populations (19–20%) and relatively lower in Trombay, India (12%), while two populations were uninfected. The only population of *B. scutellaris* that was studied, carried Entomoplasmatales infections at medium rate (14.3%) and populations of *B. correcta*, *Z. cucurbitae*, and*Z. tau* at even lower (1.8–10%; Table 1). *Spiroplasma* infections were observed in only three individuals, two of them originating from a population of *B. dorsalis* from Trombay, in India and the third one from a population of *Z. cucurbitae* from Dinajpur, in northern Bangladesh (6.7 and 1% respectively). The total prevalence in each species was 1.1 and 0.4% (Table 2). Differences in infection rates were also observed between different countries. In *B. zonata* for instance, 14.3% of samples from India were infected with Entomoplasmatales while in Bangladesh the infection rate was calculated at 5% (Table 1).

Two populations of *B. dorsalis* and one of *Z. tau* were found to harbor*Cardinium* infections with much different prevalence. The most prevalent infection was identified in a population of*B. dorsalis* from Dinajpur, Bangladesh with 45.5% (Table 1) (chi-squared test:*p*-values< 0.01). A population of*Z. tau*, also from Dinajpur, carried a 5% infection, while the other infected *B. dorsalis* population originating from Palampur, India displayed a 6.7% infection rate. The prevalence of *Cardinium* infections was 5.8% in *B. dorsalis* and 0.9% in *Z. tau* (Table 2) (chi-squared test: *p*-values< 0.04). Finally, in the case of*B. dorsalis*, populations from Bangladesh showed higher prevalence, but without statistical significance, than those from India (6.9% compared to 2.2%).

### MLST genotyping for Wolbachia strains

Sequence analysis revealed the presence of several alleles for all MLST, *wsp* and 16S *rRNA* loci: three for *gatB*, two for *coxA*, two for *hcpA*, two for *ftsZ*, two for*fbpA*, two for *wsp* and nine for the 16S *rRNA*. Interestingly, more than half of the MLST and *wsp* alleles were new in the *Wolbachia* MLST database: two for *gatB*, one for *coxA*, one for*hcpA*, two for *ftsZ*, one for *fbpA* and one for *wsp*, respectively (Table 3). Cloning and sequencing of the MLST, *wsp* and 16S *rRNA* gene amplicons clearly indicated the presence of multiple strains within individuals of three populations (Table 3). In more detail, multiple bacterial strains with two potential Sequence Types (STs, combination of alleles) were detected in the infected *B. zonata* sample (2.2) from Trombay. The second infected *B. zonata* sample (8.2) contained four possible ST combinations. In addition to these multiple infections, we found double 16S *rRNA* alleles in four Indian samples, in *B. correcta* (1.4 and 01.5H) from Trombay, in *B. scutellaris* (02.5E) from Palampur and in *B. zonata* (01.4E) from Raichur.

**Table 3:** *Wolbachia* MLST,*wsp*, 16S *rRNA* allele profiles and pseudogenes for infected *Bactrocera* and *Z. cucurbitae* populations

			*Wolbachia* MLST							
Sample code	species	Country, Area	ST	*gatB*	*coxA*	*hcpA*	*ftsZ*	*fbpA*	*wsp*	16S *rRNA*
03.7D	*B. dorsalis*	Bangladesh, Rajshahi	–	–	–	–	–	–	–	AL4 + PW
03.3B	*B. zonata*	Bangladesh, Rajshahi	–	–	–	–	–	–	–	PW
BC.18	*Z. cucurbitae*	Bangladesh, Dinajpur	–	–	–	–	–	–	–	PW
BC.27										
1.4	*B. correcta*	India, Maharashtra, Trombay	*w*Bco	8	New1	103	New1	160	335	AL2 + AL9 + PW
01.5H			–	–	–	–	–	–	–	AL5 + AL7
DD2.2	*B. dorsalis*	India, Maharashtra, Trombay	*w*Bdo	New2	New1	New1	New2	New1	New1	AL1
01.10B			–	–	–	–	–	–	–	AL2
01.11A			–	–	–	–	–	–	–	AL2
02.11D	*B. dorsalis*	India, Himachal Pradesh, Palampur	–	–	–	–	–	–	–	AL3 + PW
02.10G	*B. nigrofemoralis*	India, Himachal Pradesh, Palampur	–	–	–	–	–	–	–	PW
02.5E	*B. scutellaris*	India, Himachal Pradesh, Palampur	–	–	–	–	–	–	–	AL2 + AL8
01.4E	*B. zonata*	India, Karnataka, Raichur	–	–	–	–	–	–	–	AL2 + AL8
2.2	*B. zonata*	India, Maharashtra, Trombay	Multi *w*Bzo-1 *w*Bzo-2	8 + New1	84	103	New1 + PW	160	335 + PW	AL6 + PW
8.2			Multi *w*Bzo-1 *w*Bzo-2 *w*Bzo-3 *w*Bzo-4	8 + New1	84 + New1	103	New1 + PW	160	335	AL3 + PW

PW: pseudogenized (with deletions) *Wolbachia* genes

New: new alleles based on MLST data

Multi: multiple potential combinations/ST of alleles

### Phylogenetic analysis

**Fig. 1:**
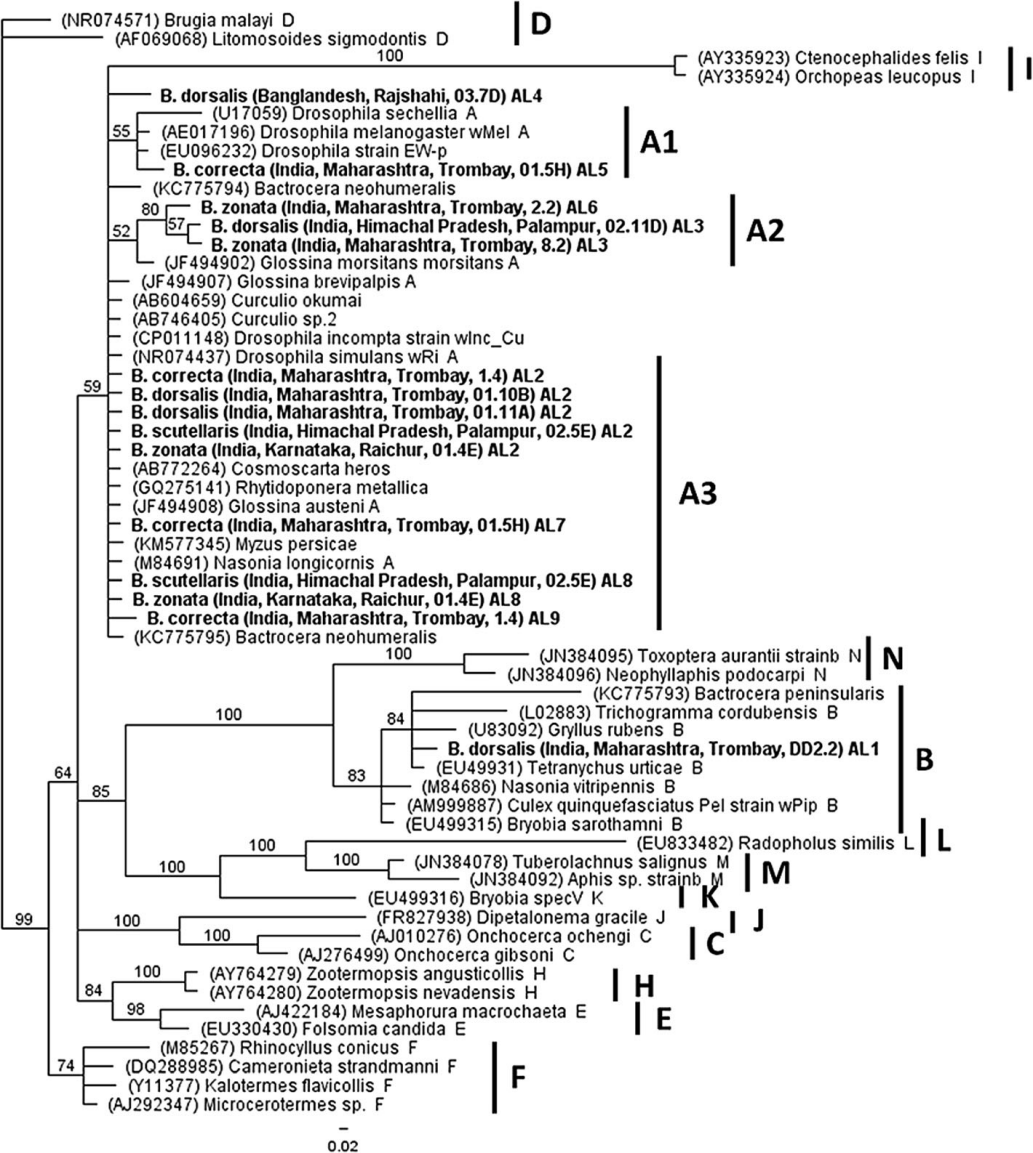
Bayesian inference phylogeny based on the 16S *rRNA* gene sequence (438 bp). The 15*Wolbachia* strains present in *Bactrocera* and indicated in bold letters (including 9 Alleles: AL1 to AL9) along with the other strains represent supergroups A, B, C, D, E, F, H, I, J, K, L, M and N. Strains are characterized by the names of their host species and their GenBank accession number. *Wolbachia* supergroups are shown to the right of the host species names. Bayesian posterior probabilities based on 1000 replicates are given (only values > 50% are indicated; *Brugia malayi* used as outgroup)

**Fig. 2:**
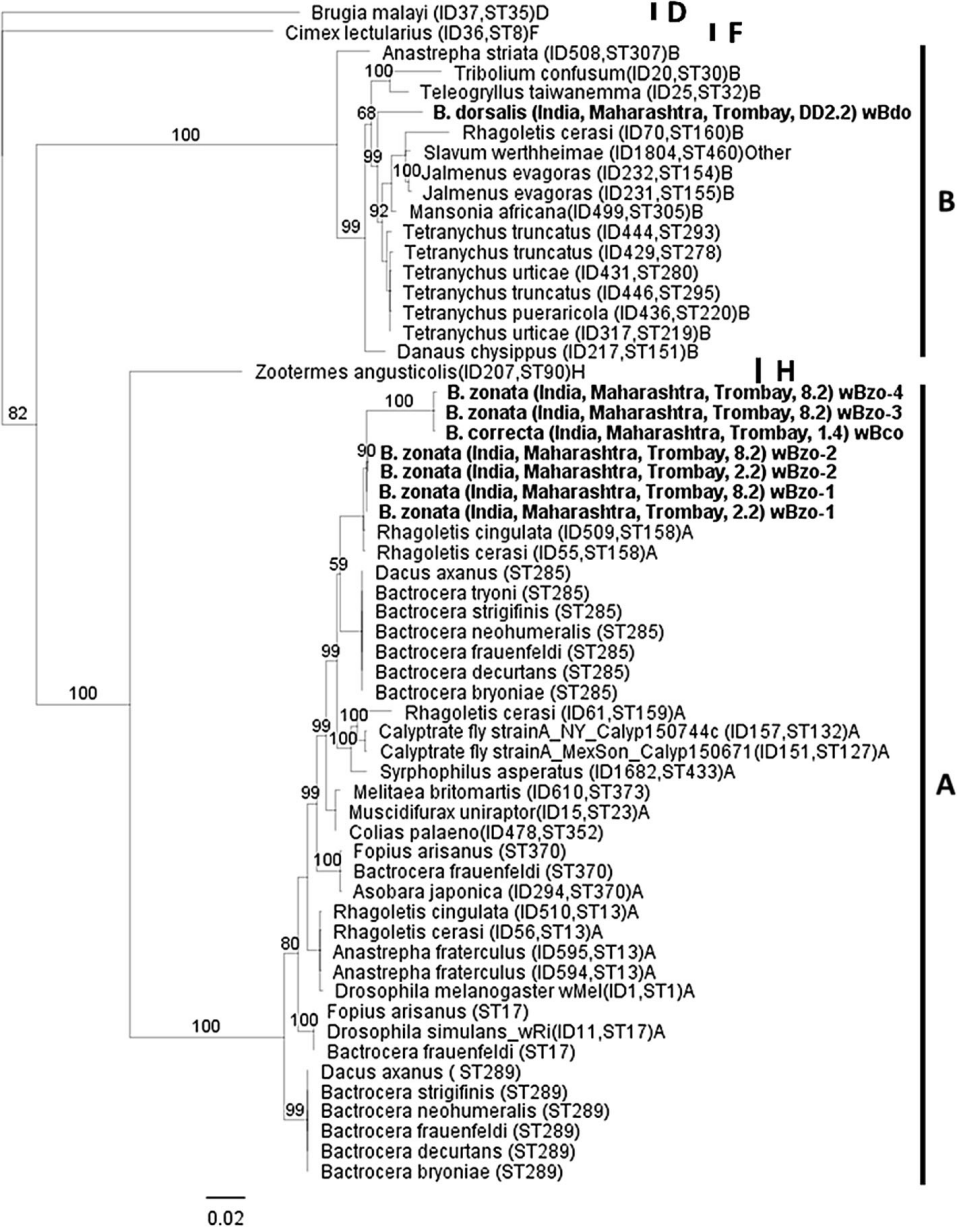
Bayesian inference phylogeny based on the concatenated MLST data (2079 bp). The eight *Wolbachia* strains present in *Bactrocera* are indicated in bold letters, while all the other strains represent supergroups A, B, D, F and H. Strains are characterized by the names of their host species and ST number from the MLST database. *Wolbachia* supergroups are shown to the right of the host species names. Bayesian posterior probabilities based on 1000 replicates are given (only values > 50% are indicated; *Brugia malayi* used as outgroup)

The *Wolbachia* phylogenetic analysis was carried out on seven *Wolbachia*-infected natural populations and was based on the datasets of all MLST (*gatB*, *coxA*, *hcpA*, *ftsZ* and *fbpA*) and 16S *rRNA* loci. Phylogenetic analysis, based on the 16S *rRNA* gene sequences, revealed that the clear majority of the *Wolbachia* strains infecting *Bactrocera* species belonged to supergroup A, except for the strain found in *B. dorsalis* sample DD2.2 from Trombay that fell into supergroup B (Fig. 1). In more detail, based on the 16S *rRNA* loci, *Wolbachia* strains infecting *Bactrocera* species classified into three clusters in supergroup A and one cluster in supergroup B (Fig. 1). The first cluster (A1) includes a *Wolbachia* strain infecting a *B. correcta* sample (01.5H) from Trombay which groups with the strain present in *Drosophila melanogaster*. The second cluster (A2) is comprised of strains present in samples from India, such as *B. dorsalis* from Palampur and *B. zonata* from Trombay which are similar to *Wolbachia* from *Glossina morsitans morsitans*. The third cluster (A3) is the largest and contains strains present in samples of *B. correcta* (Trombay), *B. dorsalis* (Trombay), *B. scutellaris* (Palampur) and *B. zonata* (Raichur) from India as well as in samples of *B. dorsalis* from Bangladesh (Rajshahi), that are closely related to *Wolbachia* strains found in *Drosophila simulans* and *Glossina austeni*. Finally, the *Wolbachia* strain infecting sample DD2.2 of *B. dorsalis* from Trombay, which fell in supergroup B, clusters with the strain from *Tetranychus urticae*. The same results were also acquired with the phylogenetic analysis based on the concatenated sequences of the MLST genes (Fig. 2). More specifically: (a) the *Wolbachia* strains*w*Bzo-3, *w*Bzo-4 (multiple infections in sample 8.2 of *B. zonata* from Trombay) and *w*Bco (infecting *B. correcta* from Trombay) were classified into a distinct cluster of supergroup A, while the*Wolbachia* strains *w*Bzo-1 and *w*Bzo-2 infecting both *B. zonata* samples from Trombay (2.2 and 8.2) were assigned into another cluster of supergroup A, (b) the strain*w*Bdo infecting *B. dorsalis* from Trombay was assigned to supergroup B. The most closely related *Wolbachia* strains to*w*Bzo-1 and *w*Bzo-2 have been detected in *Rhagoletis cingulata* (ST 158) and *Rhagoletis cerasi* (ST 158) (Fig. 2).

**Fig. 3:**
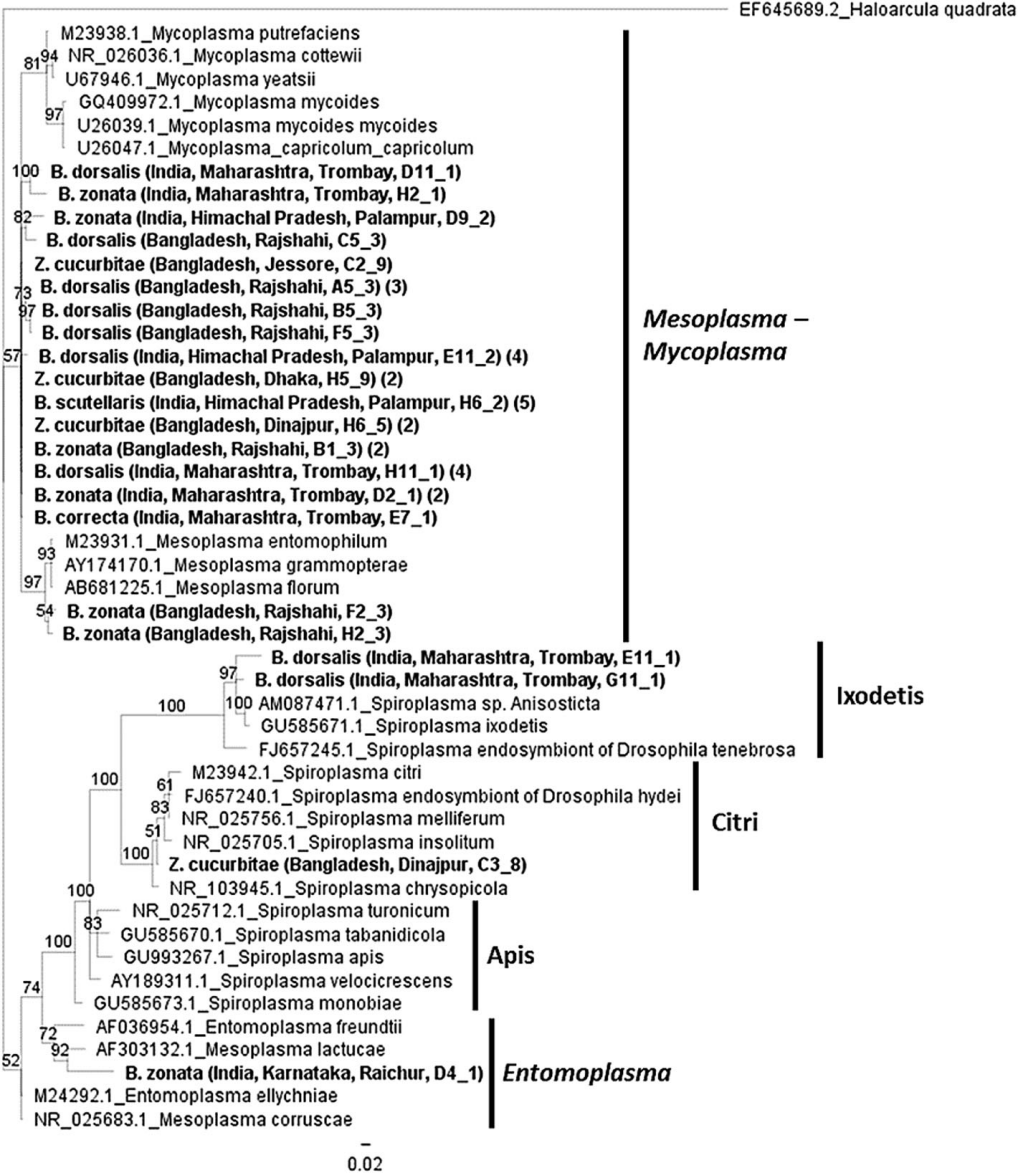
Bayesian inference phylogeny based on the Entomoplasmatales 16S *rRNA* gene sequence (301 bp). The strains present in *Bactrocera* and *Z. cucurbitae* are indicated in bold letters. Most samples represent the *Entomoplasma* and *Mesoplasma-Mycoplasma* groups while three sequences represent the Ixodetis and Citri groups of *Spiroplasma*. The Ixodetis, Citri and Apis clades are shown to the right of the *Spiroplasma* species names. Bayesian posterior probabilities based on 1000 replicates are given (only values > 50% are indicated; *Haloarcula quadrata* used as outgroup). For each strain, their GenBank accession number is also given on the left. Two sequences were removed due to short length (one from*B. dorsalis* and one from*Z. tau*). Parentheses on the right of the name indicate number of sequences from that population

**Fig. 4:**
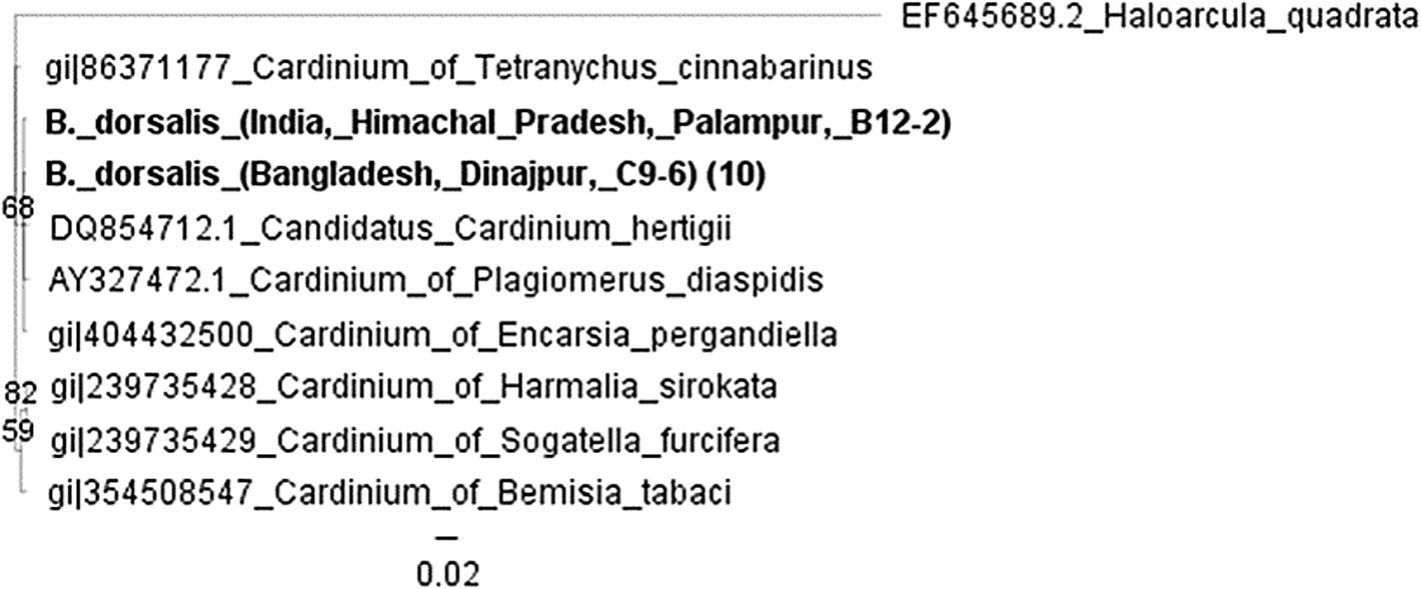
Bayesian inference phylogeny based on the *Cardinium* 16S *rRNA* gene sequence (354 bp). The strains present in *Bactrocera* are indicated in bold letters. The 11 sequences from *B. dorsalis* and one from *Z. tau* (removed due to shorter length) group with *Cardinium* sequences found in *Encarsia pergandiella* and *Plagiomerus diaspidis*. Bayesian posterior probabilities based on 1000 replicates are given (only values > 50% are indicated; *Haloarcula quadrata* used as outgroup). For each strain, their GenBank accession number is also given on the left. Parentheses on the right of the name indicate number of sequences from that population

Phylogenetic analysis based on the 16S *rRNA* gene revealed that the majority of the Entomoplasmatales infecting *Bactrocera* and *Zeugodacus* species clustered with *Mesoplasma corruscae* and *Entomoplasma ellychniae* (Fig. 3). These 32 sequences were found in populations of *B. correcta*, *B. dorsalis*, *B. scutellaris* and*B. zonata* from various regions of India and in populations of *B. dorsalis*, *B. zonata* and *Z. cucurbitae* from Bangladesh. Two sequences from *B. zonata* samples (Rajshahi) grouped with the closely related *Mesoplasma entomophilum* cluster. One sequence from *B. zonata* (Raichur) clustered with *Mesoplasma lactucae*, in the closely related *Entomoplasma* group. A strain found in *Z. cucurbitae* from Bangladesh (Dinajpur) was clustered with the *Spiroplasma citri-chrysopicola-mirum* group and two strains found in a population of *B. dorsalis* from the area of Trombay in India, fell into the *Spiroplasma ixodetis* group. Finally, the phylogenetic analysis of *Cardinium* 16S *rRNA* sequences that were identified in two populations of*B. dorsalis* (Dinajpur and Palampur) were grouped with *Cardinium* species infecting*Encarsia pergandiella* and *Plagiomerus diaspidis* that compose group A of*Cardinium* strains (Fig. 4).

### Detection of Wolbachia pseudogenes

**Fig. 5:**
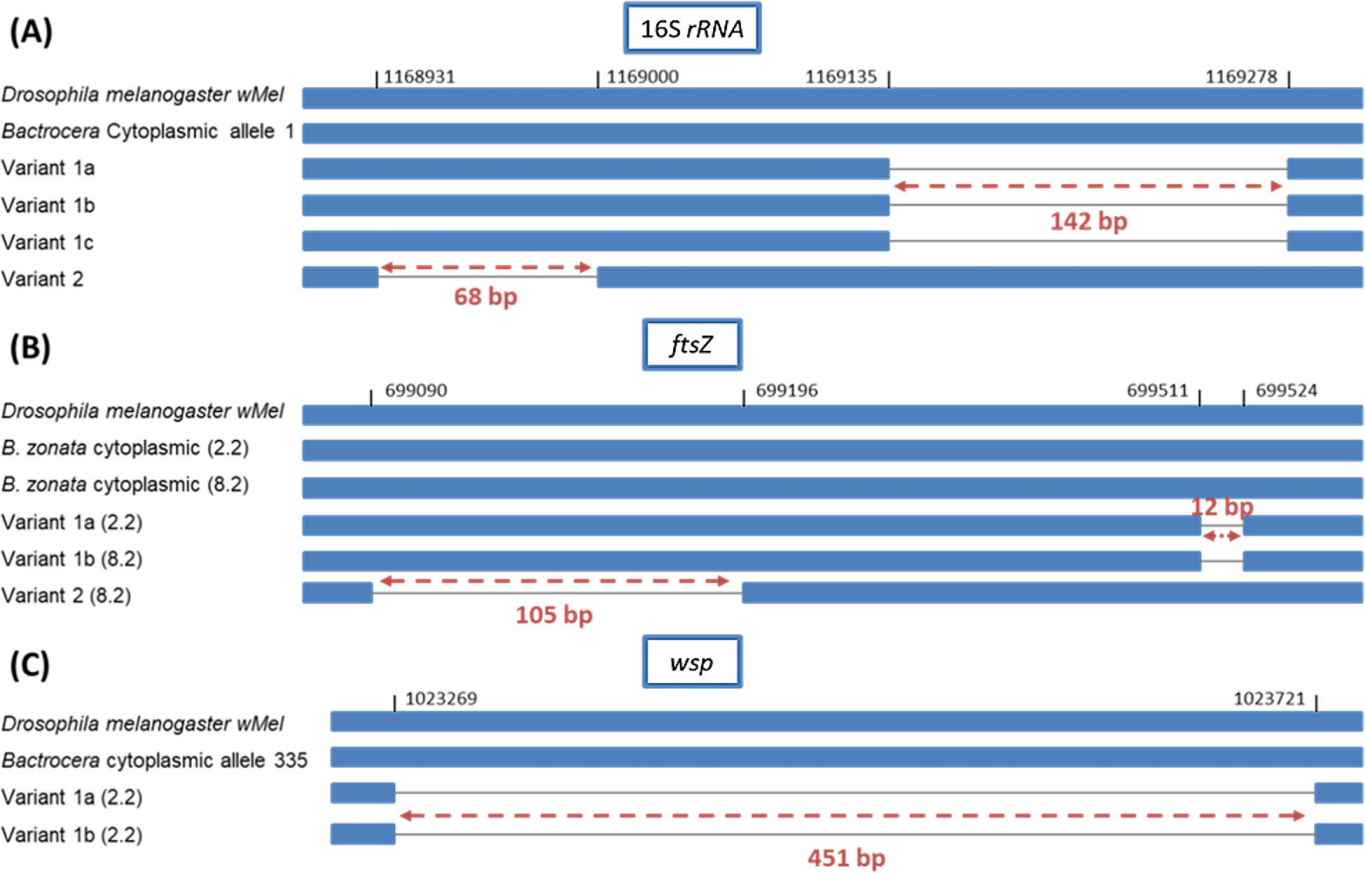
Overview of three *Wolbachia* pseudogenes carrying deletions of various sizes. The 16S *rRNA*,*ftsZ* and *wsp* gene fragments of *Wolbachia* chromosomal insertions sequenced from natural *Bactrocera* and *Zeugodacus* populations aligned with the corresponding regions of strain *w*Mel and *Wolbachia* strains infecting *Bactrocera* flies (cytoplasmic). Grey lines represent the deletion region. The black numbers show the positions before and after the deletions in respect to the*w*Mel genome. The red arrows and numbers indicate the size of deletion in base pairs. Variants exhibit small number of SNPs. **a**Variant 1a: *B. zonata* (Bangladesh, Rajshahi, 03.3B), *B. correcta* (India, Trombay, 1.4), *B. dorsalis* (India, Palampur, 02.11D), *B. nigrofemoralis* (India, Palampur, 02.10G),*B. zonata* (India, Trombay, 2.2). Variant 1b:*B. dorsalis* (Bangladesh, Rajshahi, 03.7D), *B. dorsalis* (India, Palampur, 02.11D), *B. zonata* (India, Trombay, 8.2). Variant 1c: *B. correcta* (India, Trombay, 1.4).Variant 2: *Z. cucurbitae* (Bangladesh, Dinajpur, 07.10H). **b** Deletions in the *ftsZ* gene were identified in two *B. zonata* samples, *B. zonata* (India, Trombay, 2.2) and *B. zonata* (India, Trombay, 8.2). Sample 8.2 carried two different types of deletions. (**C**) *B. zonata* (India, Trombay, 2.2) contained *wsp* pseudogenes with two different types of deletions

The presence of two distinct PCR amplification products was observed for the 16S *rRNA* gene in samples from four *Bactrocera* populations during the*Wolbachia*-specific 16S *rRNA*-based screening (Table 3). The first product had the expected 438 bp size while the second was 296 bp (Fig. 5a). Interestingly, the populations of *B. nigrofemoralis* from Palampur, India and *B. zonata* from Rajshahi, Bangladesh were found to contain only the smaller pseudogenized sequence. On the contrary, other samples from India including, *B. correcta* (sample 01.5H) and*B. dorsalis* from Trombay, *B. scutellaris* from Palampur and *B. zonata* from Raichur, contained only the expected 438 bp fragment (Table 3). When sequenced, both PCR products appeared to be of *Wolbachia* origin. The 438 bp product corresponded to the expected 16S *rRNA* gene fragment, while the shorter product contained a deletion of 142 bp (Fig. 5a). The 296 bp short version of the gene was detected in seven individuals from various *Bactrocera* species, including *B. correcta, B. dorsalis*,*B. nigrofemoralis* and *B. zonata*. Three different types of deletions were found, with minor changes in their nucleotide sequence compared to the cytoplasmic *Wolbachia* 16S *rRNA* gene fragment found in *Drosophila melanogaster* and various *Bactrocera* species in this study (Fig. 5a). *Zeugodacus cucurbitae* from Dinajpur, Bangladesh contained only pseudogenized *Wolbachia* 16S *rRNA* gene sequences. In this case, however the deletion was only 68 bp and the resulting pseudogene had a size of 370 bp (Fig. 5a). The presence of distinct amplicons was also observed during*Wolbachia* MLST analysis for genes*ftsZ* and *wsp*. In both cases, apart from the expected PCR product, a smaller fragment was also detected (Fig. 5b, c). Multiple *ftsZ* gene products were found in two samples (2.2 and 8.2) belonging to the population of*B. zonata* from Trombay, India. Two different short amplicons were observed. Sequence analysis revealed that the large product had the expected size of 524 bp while the short ones were either 512 bp or 419 bp long (Fig. 5b). The 512 bp fragment contained a small deletion of 12 bp while the 419 bp one, a much larger of 105 bp. The 419 bp fragment was only detected in sample 8.2. In the case of the 512 bp fragment, two different variants were found with minor changes in their sequence (Fig. 5b). Two distinct PCR products were also observed during amplification of the *wsp* gene in sample 2.2 of *B. zonata* from India (Trombay) (Fig. 5c). After sequence analysis, the larger product appeared to have the expected 606 bp size while the second was significantly smaller, consisting of only 155 bp. Two such pseudogenes were found in this case, with minor differences in their sequence (Fig. 5c).

## Discussion

In this study, *Wolbachia*, Entomoplasmatales and *Cardinium* infections were identified in several *Bactrocera* and *Zeugodacus* species. Interestingly, none of the examined populations contained sequences belonging to *Arsenophonus*.

### Infections prevalence

The prevalence of *Wolbachia* infections was found to vary between different species. For the first time, infections were detected in *B. scutellaris* and *B. zonata*. In the case of *B. correcta*, a previous study on wild samples from Thailand reported a higher infection rate (50%) than the one observed in our work (33%), but was based on only two screened individuals [94]. Contrary to the infection rate we detected in *B. dorsalis* (13.2%), most wild and laboratory populations examined up to date, were found to harbor no*Wolbachia* infections [94–96]. However, there are two cases of active*Wolbachia* infections that have been reported in *B. dorsalis* from Thailand. One is a low rate infection (0.9%; 2 individuals out of 222) and the other shows medium prevalence (50%) but is based only on one infected sample [94]. On the other hand, no *Wolbachia* infections were present in *B. minax*, *B. nigrofemoralis*, *D. longicornis*, *Z. cucurbitae* and*Z. tau*. It is noteworthy that previous studies reported infections, but overall with very low prevalence, in *Z. cucurbitae* (4.2%) and *Z. tau* (1%) [94]. Recently, *Wolbachia* endosymbiont of *Culex quinquefasciatus* Pel was detected as the dominant species, with ~ 98% prevalence, in all the life stages studied in samples of *B. latifrons* (Hendel) from Malaysia using next-generation sequencing [97]. This occurrence is notably higher than any other*Bactrocera* species originating from Southeast Asia and Oceania.

Most of the *Wolbachia*-infected populations were found in India, in areas located in the far North (Palampur), close to the West coast (Trombay) as well as in the South (Raichur). Only one infected population was detected in Bangladesh, close to the city of Rajshahi, on the western border with India. In the case of *B. zonata*, the presence of *Wolbachia* decreased and eventually the infection was lost as we moved towards the North and away from the equator. Otherwise, this trend could mean that the infection is currently spreading from South to North. At the same time, infections in *B. dorsalis* exhibited the exact opposite behavior. The low prevalence infection detected in the population originating from Rajshahi, in western Bangladesh, close to the border with India, could be the result of a current spreading from the neighboring infected Indian populations. No individuals from Raichur were screened, so the picture of the infection in *B. dorsalis* further to the South is incomplete. Infected populations of *B. correcta* followed a similar pattern to *B. dorsalis*. In this case, however, no population from Northern India (Palampur) was included in the screen. Finally, it was impossible to determine a trend in the case of *B. scutellaris* since the only infected population was found in the North of India (Palampur).

Low density (< 10%) Entomoplasmatales infections were detected in multiple *Zeugodacus* and *Bactrocera* species. Previous screenings of laboratory populations of five *Bactrocera* species did not reveal any infections with members of the Entomoplasmatales [95]. *Spiroplasma* infections, the only genus within the order with species known to induce reproductive phenotypes, were identified in *B. dorsalis* and *Z. cucurbitae* with much lower frequencies (~ 1%) compared to other fly species belonging to the genera of *Drosophila* (0–53%) [38, 98]*Glossina* (5.8–37.5%) [75] and *Phlebotomus* (12.5%) [99]. The geographical distribution of infected populations appeared to be widespread in various areas of Bangladesh and India. In both*B. dorsalis* and *B. zonata*, subtropical and tropical populations were generally characterized by similar infection rates with little fluctuation, suggesting that geography does not influence the dispersion of infections. For the remaining fruit fly species infected with Entomoplasmatales, we could not extract any useful information about the geographical distribution of infections either due to the presence of only one infected population or due to the proximity of infected populations.

Populations infected with *Cardinium* originated only from subtropical regions and harbored either medium or low prevalence infections. Previously, 244 species of flies belonging to the Empidoidea (Order: Diptera), which consists of four families such as the long-legged flies (Family: Dolichopodidae) and the dance flies (Family: Hybotidae), were found to contain *Cardinium* infections in only ten species, with an incidence rate of 4% [28]. A similar study in various arthropods did not identify any *Cardinium* sequences in the seven families of Diptera studied [33] while laboratory populations of various *Bactrocera* species were also free of *Cardinium* infections [95]. However, higher occurrence of *Cardinium* was identified in*Culicoides* biting midge species (Diptera: Ceratopogonidae) with infection rates reaching up to 50.7, 72 or 100% [80, 100]. It seems that a wide range of*Cardinium* infections can be found in different fly species.

### Genotyping - phylogeny

The 16S *rRNA*, MLST and *wsp*-based sequence analysis results are in accordance with a previous study that was based on 16S *rRNA* and *wsp* phylogeny, in which *Wolbachia* strains infecting various*Bactrocera* species from Australia, like*B. bryoniae* (Tryon), *B. decurtans* (May), *B. frauenfeldi* (Schiner) and *B. neohumeralis* (Hardy), were clustered in supergroup A [96]. Another study, based on the *ftsZ* and *wsp* genes, identified strains belonging to both supergroups A and B, in samples from Thailand from various species including, *B. ascita* (Hardy), *B. diversa* (Coquillett) and *B. dorsalis* [101], even though a previous work on the same samples found strains belonging mostly to supergroup B, except for those found in *B. tau* (now *Z. tau*) that belonged to supergroup A [94]. The phylogenetic analysis based on the 16S *rRNA* gene sequence revealed the presence of closely related *Wolbachia* strains in different *Bactrocera* species (Fig. 1), which could be the result of horizontal transmission between insect species, as has been previously reported in the case of the parasitic wasp genus *Nasonia* and its fly host*Protocalliphora* [102] as well as in other insects [70, 103–105]. In addition, populations of various species, including *B. correcta*, *B. dorsalis*,*B. scutellaris* and *B. zonata* from different locations harbor very closely related *Wolbachia* strains, suggesting that the geographical origin of their hosts did not lead to *Wolbachia* strain divergence. However, some divergence was observed between samples of the same species (e.g. *B. correcta*) from the same population (Trombay; subgroups A1, and A3), and between different populations of a species (e.g.*B. zonata*; Trombay and Raichur; A2 and A3 respectively). Distantly related *Wolbachia* strains were seen between different *B. dorsalis* populations, but also in samples from the same population (Trombay, A3 and B). Strains belonging to supergroups A and B have been previously found to occur in the same species [102, 106]. The same picture, with closely related strains between different species and a distantly related strain from *B. dorsalis* from Trombay, was also seen in the MLST/wsp based phylogeny. Some degree of divergence was also observed between *B. zonata* samples of the same population (Trombay) similar to the one observed in the 16S *rRNA* gene-based phylogeny.

Phylogenetic analysis on the 16S *rRNA* gene sequences revealed that most Entomoplasmatales strains grouped with the closely related species *Mesoplasma corruscae* and *Entomoplasma ellychniae*. Overall, three samples were found to carry *Spiroplasma* infections. Two of the 16S *rRNA* gene sequences were classified into the ixodetis group and one into the citri-chrysopicola-mirum group. *Spiroplasma* strains infecting tsetse flies were also clustered in the citri-chrysopicola-mirum group [75]. On the other hand, *S. ixodetis* is mostly found in ticks [107–109]. All*Cardinium* strains described in this study were similar to the strain infecting the parasitic wasp *Encarsia pergandiella* (Order: Hymenoptera). Similar strains were also found in other parasitic wasps of the genus *Encarsia* as well as in armored scale insects (Order: Hemiptera) like *Aspidiotus nerii* and *Hemiberlesia palmae* [37].

### Wolbachia pseudogenes

In the present study, three *Wolbachia* genes, 16S *rRNA*,*ftsZ* and *wsp*, were found to harbor deletions of various sizes in their sequence. The most common pseudogenes were identified in the case of the 16S*rRNA* gene, in four *Bactrocera* species and *Z*. *cucurbitae* (Fig. 5a) while shorter copies of the*ftsZ* and *wsp* genes were found only in *B. zonata*. It is worth mentioning that pseudogenized sequences were found both in populations that harbored presumably active *Wolbachia* infections and in uninfected ones. Interestingly, the 16S *rRNA* and *ftsZ* pseudogenes were similar to those described previously in *Glossina* species [86], which were shown to be incorporated in the host genome. The similarity in sequence with the *Glossina* pseudogenes, along with the lack of amplification of all marker genes (MLST and *wsp*), could suggest that the identified pseudogenes may be integrated into the genome of*Bactrocera* flies. *Wolbachia* pseudogenes (16S *rRNA*, *wsp*, *coxA*, *hcpA* and*fbpA*) have been previously identified in two *Bactrocera* species (*B. peninsularis* (Drew & Hancock) and *B. perkinsi*) from tropical Australian populations with amplification results also suggesting horizontal gene transfer to the host genome [96]. Even though horizontal gene transfer is much more common between prokaryotes, many cases have been described between endosymbiotic bacteria and their insect hosts [82]. These interactions may have significant impact on the genomic evolution of the invertebrate hosts. Pseudogenized*Wolbachia* sequences and horizontal transfer events have been reported in various *Wolbachia*-infected hosts [83–86, 89, 90, 92, 93]. It is worth noting that in some cases horizontally transferred *Wolbachia* genes are expressed from the host genome, as reported in the mosquito *Aedes aegypti* and in the pea aphid *Acyrthosiphon pisum* [89, 92, 93].

## Conclusions

*Wolbachia*, *Cardinium*, *Spiroplasma* and its close relatives, *Entomoplasma* and *Mesoplasma*, are present in wild populations of*Bactrocera* and *Zeugodacus* species from Southeast Asia. Strain characterization and phylogenetic analyses were performed primarily with the 16S *rRNA* gene and additionally, in the case of *Wolbachia*, with the *wsp* and MLST gene markers, revealing the presence of supergroup A and B *Wolbachia* strains along with new and previously identified *Wolbachia* MLST and *wsp* alleles, *Spiroplasma* strains belonging to the citri-chrysopicola-mirum and ixodetis groups as well as sequences clustering with *Mesoplasma* and *Entomoplasma* species, and finally group A *Cardinium* species similar to those infecting *Encarsia pergandiella* and *Plagiomerus diaspidis*. Even though the geographical map of infections is incomplete, it seems that *Wolbachia* are more common in Indian populations and possibly spreading to neighboring countries, while Entomoplasmatales infections are widespread in both Indian and Bangladeshi populations. Fruit flies infected with these bacterial taxa were found in both tropical and subtropical regions. On the other hand, *Cardinium* infections were less common and were only found in subtropical populations. The detection of *Wolbachia* pseudogenes, containing deletions of variable size, implies putative events of horizontal gene transfer in the genome of the tephritid fruit fly populations studied which could be remnants of past infections. Further study of additional species and wild populations could provide a more detailed report of the infection status for these specific endosymbiotic bacteria that may function as reproductive parasites. The detailed characterization of existing strains could shed more light on the host-symbiont interactions, which could be potentially harnessed for the enhancement of the sterile insect technique (SIT) and related techniques as components of area-wide integrated pest management (AW-IPM) strategies for the control of insect pest populations.

## Methods

### Sample collection, preparation and DNA extraction

**Fig. 6:**
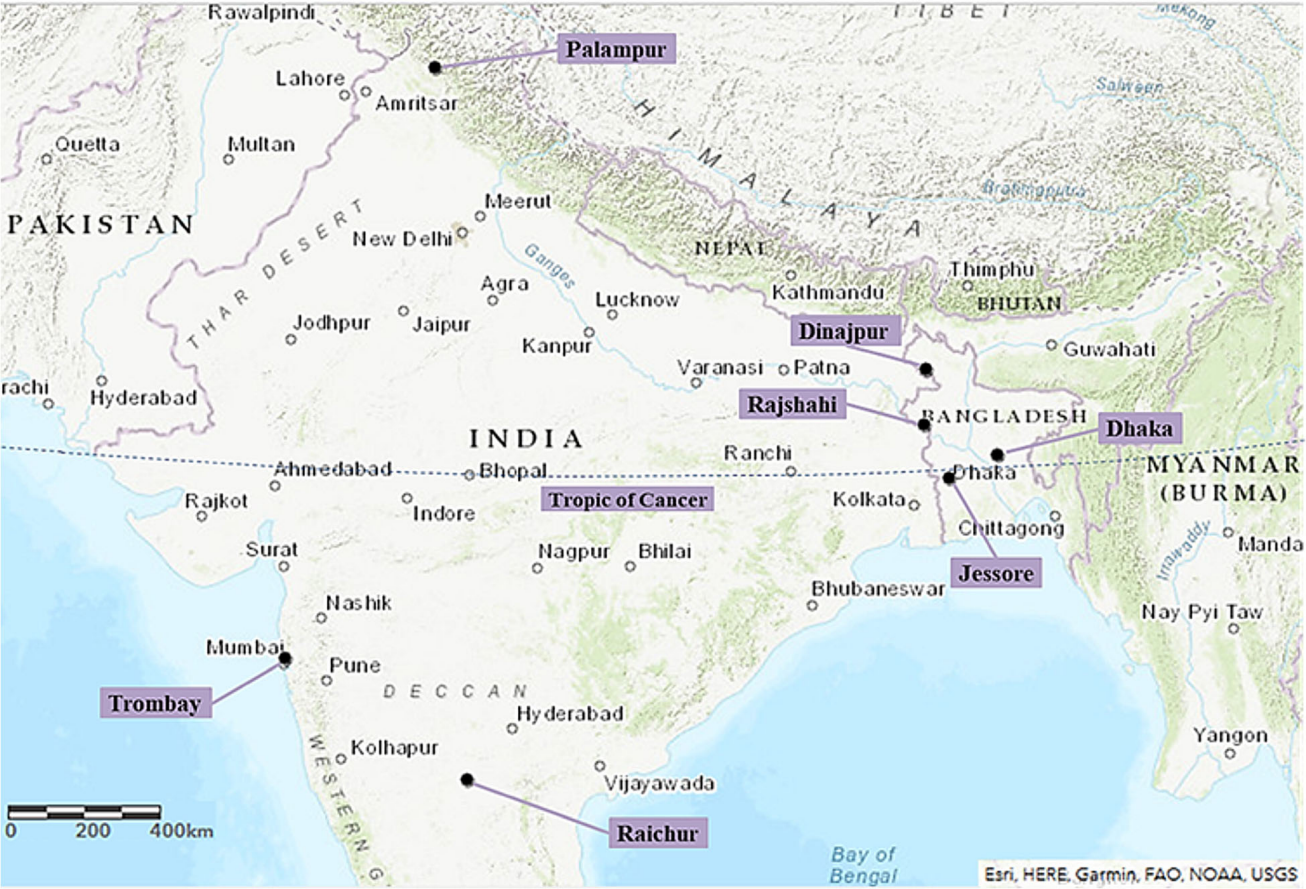
Map showing tropical (south of the Tropic of Cancer (dotted line)) and subtropical (north) sampling locations in Bangladesh and India (created with ArcGIS, by Esri)

Analyzed samples belonged to nine species of fruit flies from three different Tephritidae genera: *Bactrocera*,*Dacus* and *Zeugodacus*. A total of 801 adult male fruit flies were collected from 30 natural populations originating from various regions of Bangladesh, China and India and stored in absolute ethanol Fig. 6 (Table 1). DNA extraction was performed immediately after the arrival of the samples in the laboratory of Molecular Genetics and Microbiology at the University of Patras. Total DNA was extracted from the whole body of adult flies using the NucleoSpin® Tissue kit (Macherey-Nagel GmbH & Co. KG) following the manufacturer’s instructions. Prior to extraction, the insects were washed with sterile deionized water to remove any traces of ethanol. Each sample contained one fly (*n* = 1). Extracted DNA was stored at − 20 °C.

### PCR screening and Wolbachia MLST

The presence of reproductive symbiotic bacteria that belong to the genera *Wolbachia*, *Spiroplasma* (and the other two genera of the Entomoplasmatales,*Entomoplasma* and *Mesoplasma*), *Cardinium* and*Arsenophonus* in natural populations of tephritid fruit flies was investigated with a 16S *rRNA* gene-based PCR assay. A fragment of variable size (301–600 bp) was amplified with the use of specific primers for each bacterial genus (Additional file 2). In the case of *Wolbachia* strains, the specific 16S*rRNA* PCR assay that was employed was described previously [86]. Prior to screening, the mitochondrial 12S *rRNA* gene was used as positive control for PCR amplification. A 377 bp fragment of the gene was amplified in all samples tested with the primers 12SCFR and 12SCRR [110]. Also, amplification of an approximately 800 bp long fragment of host mitochondrial cytochrome oxidase I (COI) gene was carried out with primers “Jerry” and “Pat” [111] in order to perform molecular characterization of the samples tested and to confirm successful DNA extraction (Additional file 3). Amplification was performed in 20 μl reactions using KAPA Taq PCR Kit (Kapa Biosystems). Each reaction contained 2 μl of 10X KAPA Taq Buffer, 0.2 μl of dNTP solution (25 mM each), 0.4 μl of each primer solution (25 μM), 0.1 μl of KAPA Taq DNA Polymerase solution (5 U/μl), 1 μl from the template DNA solution and was finalized with 15.9 μl of sterile deionized water. For each set of PCR reactions performed, the appropriate negative (no DNA) and positive controls were also prepared. The PCR protocol was comprised of an initial denaturation step at 95 °C for 5 min, followed by 35 cycles of denaturation for 30 s at 95 °C, annealing for 30 s at the required annealing temperature (T_a_) for every pair of primers (54 °C for *Wolbachia*, 56 °C for*Arsenophonus* and *Cardinium*, 58 °C for *Spiroplasma*, 54 °C for the 12S *rRNA* gene and 49 °C for mtCOI) and extension at 72 °C for 1 min. A final extension step was performed at 72 °C for 5 min.

In order to genotype the *Wolbachia* strains present in infected specimens (Table 3), fragments of the MLST (*gatB*, *coxA*,*hcpA*, *fbpA* and *ftsZ*) and *wsp* genes were amplified with the use of their respective primers [74] (Additional file 2). Ten *Wolbachia*-infected populations (three Bangladeshi and seven Indian) were initially selected for genotyping using the MLST and *wsp* genes. Efforts were made to amplify the MLST genes in all selected samples, however, most PCRs failed, resulting in the successful amplification of all the MLST genes for only four samples (Table 3). Due to these difficulties, the characterization of the bacterial strains present in the remaining infected flies was limited to the 16S *rRNA* gene. The four samples that were amplified belonged to three *Bactrocera* species, *B. correcta*, *B. dorsalis*, and*B. zonata* (Table 3). Amplification was performed in 20 μl reactions with the following PCR mix: 2 μl of 10X KAPA Taq Buffer, 0.2 μl of dNTP mixture (25 mM each), 0.4 μl of each primer solution (25 μM), 0.1 μl of KAPA Taq DNA Polymerase solution (5 U/μl), 1 μl from the template DNA solution and 15.9 μl of sterile deionized water. PCR reactions were performed using the following program: 5 min of denaturation at 95 °C, followed by 35 cycles of 30 s at 95 °C, 30 s at the appropriate temperature for each primer pair (52 °C for*ftsZ*, 54 °C for *gatB*, 55 °C for *coxA*, 56 °C for *hcpA*, 58 °C for *fbpA* and *wsp*), 1 min at 72 °C and a final extension step of 10 min at 72 °C.

Due to products of variable size and the presence of multiple infections, we selected one representative sample from each *Wolbachia*-infected species population and cloned the PCR products of the *Wolbachia* 16S*rRNA*, *wsp* and MLST genes (Table 3) into a vector (pGEM-T Easy Vector System, Promega) according to the manufacturer’s instructions. The ligation product was used to transform DH5α competent cells, which were plated on ampicillin/X-gal selection Petri dishes. At least three clones were amplified by colony PCR [112] with primers T7 and SP6 (Thermo Fischer Scientific Inc.). Amplification was performed in 50 μl reactions each containing: 5 μl of 10X KAPA Taq Buffer, 0.4 μl of dNTP mixture (25 mM each), 0.2 μl of each primer solution (100 μM), 0.2 μl of KAPA Taq DNA Polymerase solution (5 U/μl) and 44 μl of sterile deionized water. The PCR protocol consisted of 5 min of denaturation at 95 °C, followed by 35 cycles of 30 s at 95 °C, 30 s at 53 °C, 2 min at 72 °C and a final extension step at 72 °C for 10 min.

### Sample purification and sanger sequencing

Throughout the experimental procedure, imaging of the desired amplification products was performed in a Gel Doc™ XR+ system (Bio-Rad) after loading 5 μl from each PCR reaction on 1.5% (w/v) agarose gels and separating them by electrophoresis. Purification of the PCR products was carried out with a 20% PEG, 2.5 M NaCl solution as previously described [113]. The concentration of purified PCR product was measured with a Quawell Q5000 micro-volume UV-Vis spectrophotometer. Purified PCR products were sequenced using the appropriate primers in each case (Additional file 2) while cloned*Wolbachia* PCR products were sequenced with the universal primers T7 and SP6. In this case, at least three transconjugants were sequenced as previously described [86]. A dye terminator-labelled cycle sequencing reaction was conducted with the BigDye Terminator v3.1 Cycle Sequencing Kit (Applied Biosystems). Reaction products were purified using an ethanol/EDTA protocol according to the manufacturer’s instructions (Applied Biosystems) and were analyzed in an ABI PRISM 3500 Genetic Analyzer (Applied Biosystems).

### Phylogenetic analysis

All gene sequences used in this study were aligned using MUSCLE, [114] with the default algorithm parameters, as implemented in Geneious 6.1.8 [115] and manually edited. Statistical significance of pairwise comparisons of infection prevalence between different species of fruit flies, areas or countries were calculated with chi-squared tests which were performed with R 3.5.1 [116]. The null hypothesis (*H*_*0*_) assumed that the variables (infection status between different species, areas or countries) were independent, and the significance level was equal to 0.05.*P*-values are presented in the text only for comparisons that show statistical significance. Alignments used in phylogenetic analyses were performed with MUSCLE [114] using the default algorithm parameters, as implemented in Geneious 6.1.8 [115]. Phylogenetic analyses of the 16S *rRNA* gene sequences and the concatenated sequences of the protein-coding MLST genes (*coxA, fbpA, ftsZ, gatB* and *hcpA*) were based on Bayesian Inference (BI). Bayesian analyses were performed with MrBayes 3.2.1 [117]. The evolutionary model was set to the Generalised Time Reversible (GTR) model with gamma-distributed rate variation and four gamma categories used. The parameters for the Markov Chain Monte Carlo (MCMC) method included four heated chains, with the temperature set to 0.2, which were run for 1,000,000 generations. The first 10,000 generations were discarded, and the cold chain was sampled every 100 generations. Also, posterior probabilities were computed for the remaining trees. All phylogenetic analyses were performed with Geneious [115]. All MLST, *wsp* and 16S *rRNA* gene sequences generated in this study have been deposited into GenBank under accession numbers MK045503-MK045529 and MK053669-MK053774.

## Supplementary information

**Additional file 1.** Prevalence of reproductive bacteria in tephritid fruit fly populations from Bangladesh, China and India using a 16S rRNA gene-based PCR screening approach. Red values in the heat map indicate high occurrence and blue values low. For each genus the absolute number and the percentage (in parentheses) of infected individuals are given. The last column on the right (“Total*”) indicates the total occurrence of all three Entomoplasmatales genera.

**Additional file 2.** Genes and PCR primers used.

**Additional file 3.** Bayesian inference phylogeny tree based on host mtDNA COI (~ 800 bp). Bayesian posterior probabilities based on 1000 replicates are given (only values > 50% are indicated).

## Data Availability

The datasets used and/or analyzed during the current study are available in NCBI.

## Abbreviations

AW-IPM: Area-Wide Integrated Pest Management
CFB: Cytophaga-Flavobacterium-Bacteroides
CI: Cytoplasmic Incompatibility
GTR: Generalised Time Reversible
HGT: Horizontal Gene Transfer
HVR: Hypervariable Region
MCMC: Markov Chain Monte Carlo
MLST: Multi Locus Sequence Typing
SIT: Sterile Insect Technique
ST: Sequence Type
