# Supplementary material for: Detection and characterization of bacterial endosymbionts in Southeast Asian tephritid fruit fly populations
Source: BMC Microbiol. 2019 Dec 24;19(Suppl 1):290. doi: 10.1186/s12866-019-1653-x (PMC7050614; doi:10.1186/s12866-019-1653-x)
Supplement: Supplementary file 1 — Additional file 1. Prevalence of reproductive bacteria in tephritid fruit fly populations from Bangladesh, China and India using a 16S rRNA gene-based PCR screening approach. Red values in the heat map indicate high occurrence and blue values low. For each genus the absolute number and the percentage (in parentheses) of infected individuals are given. The last column on the right (“Total*”) indicates the total occurrence of all three Entomoplasmatales genera. [file 12866_2019_1653_MOESM1_ESM.docx]

**Additional file 1**: Prevalence of reproductive bacteria in tephritid fruit fly populations from Bangladesh, China and India using a 16S *rRNA* gene-based PCR screening approach. Red values in the heat map indicate high occurrence and blue values low. For each genus the absolute number and the percentage (in parentheses) of infected individuals are given. The last column on the right (“Total*”) indicates the total occurrence of all three Entomoplasmatales genera.

|  |  |  |  |  |  |  |  | **Entomoplasmatales** | | | |
| --- | --- | --- | --- | --- | --- | --- | --- | --- | --- | --- | --- |
|  | **Species** | **Country** | **State** | **Area** | **Samples** | ***Wolbachia*** | ***Cardinium*** | ***Spiroplasma*** | ***Entomoplasma*** | ***Mesoplasma*** | **Total*** |
| 1 | *B. correcta* | India | Maharashtra | Trombay | 25 | 10 (40) | 0 | 0 | 1 (4) | 0 | 1 (4) |
| 2 | *B. correcta* | India | Karnataka | Raichur | 5 | 0 | 0 | 0 | 0 | 0 | 0 |
| 3 | *B. dorsalis* | Bangladesh | - | Rajshahi | 36 | 1 (2.8) | 0 | 0 | 6 (16.7) | 0 | 6 (16.7) |
| 4 | *B. dorsalis* | Bangladesh | - | - | 29 | 0 | 0 | 0 | 0 | 0 | 0 |
| 5 | *B. dorsalis* | Bangladesh | - | Dinajpur | 22 | 0 | 10 (45.5) | 0 | 0 | 0 | 0 |
| 6 | *B. dorsalis* | Bangladesh | - | Dhaka | 34 | 0 | 0 | 0 | 0 | 0 | 0 |
| 7 | *B. dorsalis* | Bangladesh | - | Jessore | 23 | 0 | 0 | 0 | 0 | 0 | 0 |
| 8 | *B. dorsalis* | India | Maharashtra | Trombay | 30 | 14 (46.7) | 0 | 2 (6.7) | 5 (16.7) | 0 | 7 (23.3) |
| 9 | *B. dorsalis* | India | Himachal Pradesh | Palampur | 15 | 10 (66.7) | 1 (6.7) | 0 | 5 (33.3) | 0 | 5 (33.3) |
| 10 | *B. minax* | China | - | - | 40 | 0 | 0 | 0 | 0 | 0 | 0 |
| 11 | *B. nigrofemoralis* | India | Himachal Pradesh | Palampur | 5 | 2^a^ (0) | 0 | 0 | 0 | 0 | 0 |
| 12 | *B. scutellaris* | India | Himachal Pradesh | Palampur | 35 | 15 (42.9) | 0 | 0 | 5 (14.3) | 0 | 5 (14.3) |
| 13 | *B. zonata* | Bangladesh | - | Rajshahi | 21 | 2^a^ (0) | 0 | 0 | 2 (9.5) | 2 (9.5) | 4 (19) |
| 14 | *B. zonata* | Bangladesh | - | Jessore | 33 | 0 | 0 | 0 | 0 | 0 | 0 |
| 15 | *B. zonata* | Bangladesh | - | Dinajpur | 26 | 0 | 0 | 0 | 0 | 0 | 0 |
| 16 | *B. zonata* | India | Maharashtra | Trombay | 25 | 10 (40) | 0 | 0 | 3 (12) | 0 | 3 (12) |
| 17 | *B. zonata* | India | Karnataka | Raichur | 5 | 4 (80) | 0 | 0 | 0 | 1 (20) | 1 (20) |
| 18 | *B. zonata* | India | Himachal Pradesh | Palampur | 5 | 0 | 0 | 0 | 1 (20) | 0 | 1 (20) |
| 19 | *D. longicornis* | Bangladesh | - | Dhaka | 21 | 0 | 0 | 0 | 0 | 0 | 0 |
| 20 | *Z. cucurbitae* | Bangladesh | - | Rajshahi | 47 | 0 | 0 | 0 | 0 | 0 | 0 |
| 21 | *Z. cucurbitae* | Bangladesh | - | Jessore | 55 | 0 | 0 | 0 | 0 | 1 (1.8) | 1 (1.8) |
| 22 | *Z. cucurbitae* | Bangladesh | - | - | 30 | 0 | 0 | 0 | 0 | 0 | 0 |
| 23 | *Z. cucurbitae* | Bangladesh | - | Dinajpur | 96 | 2^a^ (0) | 0 | 1 (1) | 2 (2.1) | 0 | 3 (3.1) |
| 24 | *Z. cucurbitae* | Bangladesh | - | Dhaka | 29 | 0 | 0 | 0 | 2 (6.9) | 0 | 2 (6.9) |
| 25 | *Z. tau* | Bangladesh | - | Jessore | 22 | 0 | 0 | 0 | 0 | 0 | 0 |
| 26 | *Z. tau* | Bangladesh | - | Dhaka | 6 | 0 | 0 | 0 | 0 | 0 | 0 |
| 27 | *Z. tau* | Bangladesh | - | Rajshahi | 31 | 0 | 0 | 0 | 0 | 0 | 0 |
| 28 | *Z. tau* | Bangladesh | - | Dinajpur | 20 | 0 | 1 (5) | 0 | 0 | 0 | 0 |
| 29 | *Z. tau* | India | Maharashtra | Trombay | 10 | 0 | 0 | 0 | 0 | 1 (10) | 1 (10) |
| 30 | *Z. tau* | India | Himachal Pradesh | Palampur | 20 | 0 | 0 | 0 | 0 | 0 | 0 |
| **Total** | **9** | **3** | **3** | **7** | **801** |  |  |  |  |  |  |
