# Supplementary material for: Detection and characterization of bacterial endosymbionts in Southeast Asian tephritid fruit fly populations
Source: BMC Microbiol. 2019 Dec 24;19(Suppl 1):290. doi: 10.1186/s12866-019-1653-x (PMC7050614; doi:10.1186/s12866-019-1653-x)
Supplement: Supplementary file 2 — Additional file 2. Genes and PCR primers used. [file 12866_2019_1653_MOESM2_ESM.docx]

**Additional file 2:** Genes and PCR primers used

| Bacterial Genus | Locus code  (*w*Mel) | Gene | Product | Name | Primer sequence 5´- 3´ | Gene length  (bp) | Fragment  size (bp) | References |
| --- | --- | --- | --- | --- | --- | --- | --- | --- |
| *Wolbachia* | WD_0146 | *gatB* | glutamyl-tRNA(Gln) amidotransferase, subunit B | gatB_F1  gatB_R1 | GAKTTAAAYCGYGCAGGBGTT  TGGYAAYTCRGGYAAAGATGA | 1,425^a^ | 369 | Baldo et al. 2006 |
| *Wolbachia* | WD_0301 | *coxA* | cytochrome c oxidase, subunit I | coxA_F1  coxA_R1 | TTGGRGCRATYAACTTTATAG  CTAAAGACTTTKACRCCAGT | 1,551^a^ | 402 | Baldo et al. 2006 |
| *Wolbachia* | WD_0484 | *hcpA* | hypothetical conserved protein | hcpA_F1  hcpA_R1 | GAAATARCAGTTGCTGCAAA  GAAAGTYRAGCAAGYTCTG | 741^a^ | 444 | Baldo et al. 2006 |
| *Wolbachia* | WD_0723 | *ftsZ* | cell division protein | ftsZ_F1  ftsZ_R1 | ATYATGGARCATATAAARGATAG  TCRAGYAATGGATTRGATAT | 1,197^a^ | 435 | Baldo et al. 2006 |
| *Wolbachia* | WD_1238 | *fbpA* | fructose-bisphosphatealdolase | fbpA_F1  fbpA_R1 | GCTGCTCCRCTTGGYWTGAT  CCRCCAGARAAAAYYACTATTC | 900^a^ | 429 | Baldo et al. 2006 |
| *Wolbachia* | WD_1063 | *wsp* | Outer surface protein | wsp_F1  wsp_R1 | GTCCAATARSTGATGARGAAAC  CYGCACCAAYAGYRCTRTAAA | 714^a^ | 513 | Baldo et al. 2006 |
| *Wolbachia* | WD_Wp16SA | *16S rRNA* | 16S ribosomal RNA | wspecF  wspecR | YATACCTATTCGAAGGGATAG  AGCTTCGAGTGAAACCAATTC | 1,447^a^ | 438 | Werren & Windsor 2000 |
| Entomoplasmatales | - | *16S rRNA* | 16S ribosomal RNA | 63F_CG TKSSspR | GCCTAATACATGCAAGTCGAACGG  TAGCCGTGGCTTTCTGGTAA* | 1,444 | 301 | Augoustinos et al. 2015  *Fukatsu & Nikoh 2000 |
| *Cardinium* | - | *16S rRNA* | 16S ribosomal RNA | CLO-f1  CLO-r1 | GGAACCTTACCTGGGCTAGAATGTATT  GCCACTGTCTTCAAGCTCTACCAAC | 907 | 354 | Gotoh et al. 2007 |
| *Arsenophonus* | - | *16S rRNA* | 16S ribosomal RNA | ArsF  ArsR3  ArsR5 | GGGTTGTAAAGTACTTTCAGTCGT*  CCTYTATCTCTAAAGGMTTCGCTGGATG*  CCCTAAGGCACGYYTYTATCTCTAA | 1,511 | 600 | *Duron et al. 2008  Augoustinos et al. 2015 |
| - | - | *12S rRNA* | mitochondrial 12S ribosomal RNA (host) | 12SCFR  12SCRR | GAGAGTGACGGGCGATATGT  AAACCAGGATTAGATACCCTATTAT | - | 380 | Hanner & Fugate  1997 |
| - | - | *mtCOI* | mitochondrial cytochrome oxidase I (host) | Jerry  Pat | CAACAYTTATTTTGATTTTTTGG  ATCCATTACATATAATCTGCCATA | 777 |  | Simon et al. 1994 |

^a^. With respect to the *w*Mel genome.
