# Supplementary figures and images for: Detection and characterization of bacterial endosymbionts in Southeast Asian tephritid fruit fly populations
Source: BMC Microbiol. 2019 Dec 24;19(Suppl 1):290. doi: 10.1186/s12866-019-1653-x (PMC7050614; doi:10.1186/s12866-019-1653-x)

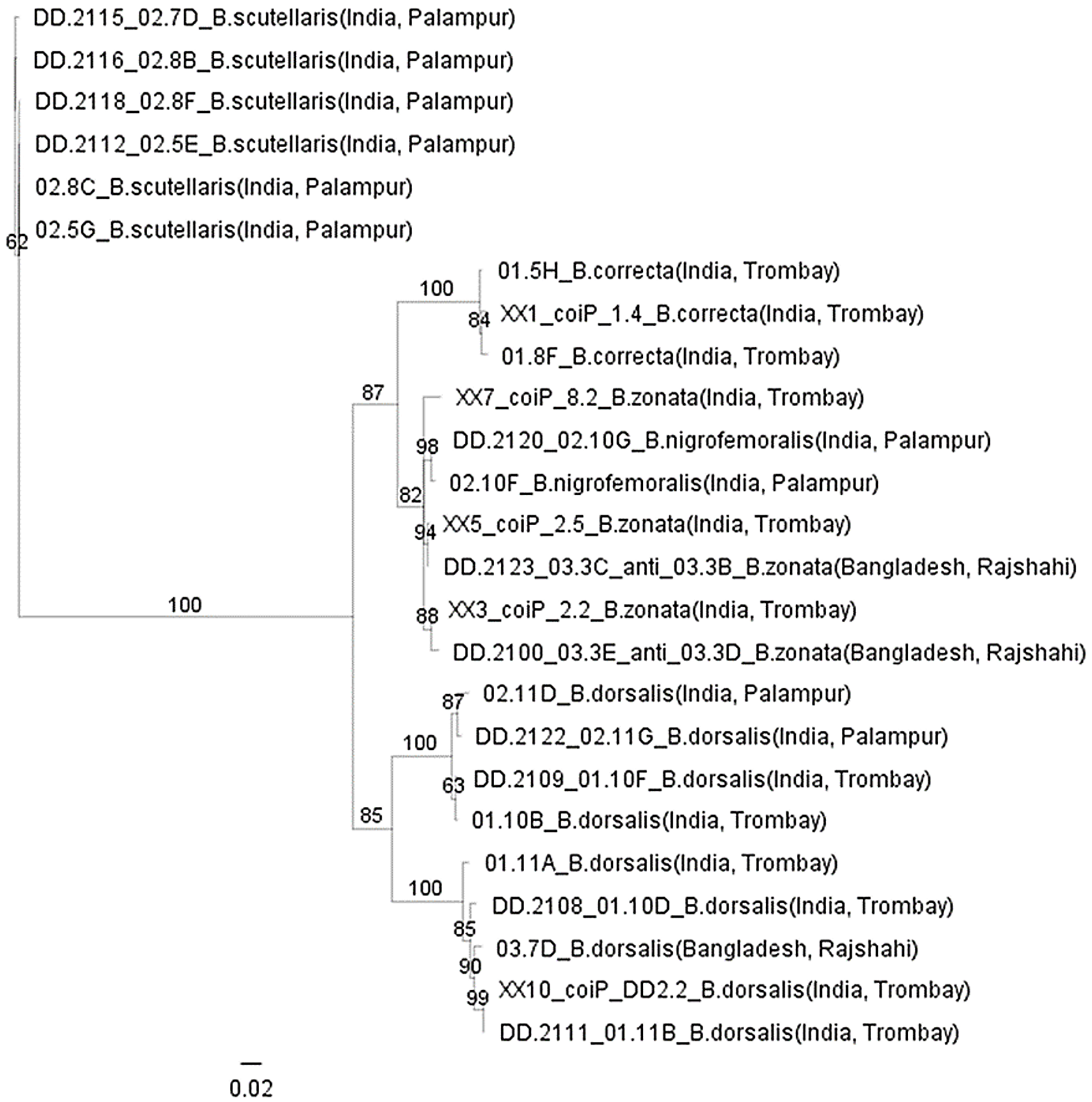

Supplement: Supplementary file 3 — Additional file 3. Bayesian inference phylogeny tree based on host mtDNA COI (~ 800 bp). Bayesian posterior probabilities based on 1000 replicates are given (only values > 50% are indicated). [file 12866_2019_1653_MOESM3_ESM.tif]
